# *Cryptococcus*: Shedding New Light on an Inveterate Yeast

**DOI:** 10.3390/jof1020115

**Published:** 2015-07-14

**Authors:** Ghady Haidar, Nina Singh

**Affiliations:** 1 Division of Infectious Diseases, Department of Medicine, University of Pittsburgh Medical Center, Pittsburgh, PA 15240, USA; E-Mail: haidarg@upmc.edu; 2 VA Pittsburgh Healthcare System, Infectious Diseases Section, and University of Pittsburgh, Pittsburgh, PA 15240, USA

**Keywords:** *Cryptococcus*, cirrhosis, immune reconstitution syndrome, transplant, human immunodeficiency virus

## Abstract

*Cryptococcus* has emerged as a significant pathogen in immunocompromised patients. While the diagnostic testing and the antifungal treatment of cryptococcal infections have become firmly established in clinical practice, new developments and areas of ambiguity merit further consideration. These include the potential for donor transmission of *Cryptococcus*; cirrhosis-associated cryptococcosis, particularly during transplant candidacy; the utility of serum cryptococcal antigen testing of asymptomatic individuals in high-prevalence, poor-resource areas; pathogenesis and treatment of the immune reconstitution syndrome, specifically in relation to antiretroviral therapy and immunosuppressive medications; and new challenges posed by the emerging species of *Cryptococcus gatti*. In this article, we summarize the literature pertaining to these topics, focusing on recent progress.

## 1. Introduction

Cryptococcosis is one of the most common opportunistic infections in immunocompromised hosts. *C. neoformans* and *C. gattii* account for ~80% and 20% of cases of human disease, respectively [1]. The global burden of cryptococcosis is estimated to be ~1 million cases with nearly 700,000 deaths annually, with most of the cases occurring in sub-Saharan Africa [1]. Indeed, *C. neoformans* is the most common cause of meningitis in this region and accounts for 20%–25% of the deaths from acquired immunodeficiency syndrome (AIDS) in Africa [2]. Cryptococcosis is also a major pathogen in solid organ transplant (SOT) recipients with an overall incidence of 2.8% (range 0.2% to 5%) [3,4]. Mortality in SOT recipients with cryptococcosis in the current era is 15% to 20% [3].

A strong cellular immune response is essential for containment of cryptococcal infections as evidenced by the fact that the vast majority of cryptococcosis occurs in patients with compromised cell-mediated immunity [5]. In addition to human immunodeficiency virus (HIV) infection and organ transplantation, major risk factors for cryptococcosis comprise liver cirrhosis, iatrogenic immunosuppression including corticosteroids and monoclonal antibodies, rheumatologic and other autoimmune diseases, idiopathic CD4+ lymphopenia, and malignancy [1,5,6]. Cryptococcosis occurs infrequently in hematopoietic stem cell transplant (HSCT) recipients [7]. According to the TRANSNET database, similar numbers of SOTs and HSCTs were performed from 2001 through 2005 (17,226 *vs.* 16,390), however cryptococcosis developed in 9% of the SOT recipients and 0% of the HSCT recipients [7]. Rarely, *C. neoformans* may occur in apparently immunocompetent patients, although selective defects in lymphocyte responsiveness to *C. neoformans* or other subtle abnormalities may account for this [6,8].

This article, although not aimed to be an exhaustive review, focuses on key areas of interest and new developments in our understanding of cryptococcal infections with implications relevant for management.

## 2. Cirrhosis-Associated Cryptococcosis

Cirrhosis has come to be recognized as a major risk factor for cryptococcosis [3,9,10]. Indeed, 21% to 36% of *Cryptococcus* infections in HIV-negative patients occur in patients with cirrhosis, and end-stage liver disease (ESLD) was the third most common predisposing factor for cryptococcosis after AIDS and iatrogenic immunosuppression [3,10].

### 2.1. Pathogenesis

Multiple defects in host immunity may account for the unique susceptibility of patients with cirrhosis to cryptococcal infections. ESLD is associated with deficiencies in circulating and ascitic fluid complement, leading to impairment in opsonization, complement-mediated inflammation and chemotaxis, antibody-mediated protection, and microbial killing by macrophages [11,12,13]. The cryptococcal polysaccharide capsule itself is a major virulence factor for this yeast, and encapsulated yeast cells are not phagocytozed nor killed as effectively as acapsular mutants [5,8]. Several other immune cell populations, such as natural killer cells, CD4+ cells, and CD8+ cells have anti-cryptococcal activity, and hyporesponsiveness of these lymphocytes occurs in cirrhotics [5,10].

It is also well-recognized that macrophages, including peritoneal macrophages, are critical in their role as antifungal effector cells [13,14]. They are involved in the production of cytokines for recruitment and activation of the host inflammatory response and are one of the first host defenses against *Cryptococcus* [13]. Peritoneal macrophages are as important as alveolar macrophages in determining the susceptibility to *Cryptococcus*, and defects may increase the risk of cryptococcosis [14]. Whether defects in peritoneal macrophage activity contribute to the susceptibility of cirrhotic patients to cryptococcal peritonitis, as has been shown for spontaneous bacterial peritonitis, remains to be determined [14].

### 2.2. Clinical Presentation

Cryptococcal disease in patients with ESLD presents as peritonitis (19%–45%), meningitis (39%–48%) and pulmonary disease (18%–37%) [9,14]. In addition, cryptococcemia and disseminated disease occur in 50% to 70% and 19% to 76% of patients with cirrhosis-related cryptococcosis, respectively, and are more likely to be associated with septic shock [3,9,10,14]. Mortality in cirrhotic patients with cryptococcosis ranges from 51% to 100%, and cirrhosis was the most significant predictor of 30-day mortality in patients with cryptococcocemia [3,9,10]. Thus, decompensated liver disease is a major risk factor for poor outcomes in those who develop cryptococcosis.

### 2.3. Transplantation in Cirrhotics with Cryptococcosis

An area where there is little clinical guidance is whether patients with cryptococcosis during transplant candidacy can safely undergo transplantation. Anecdotally, favorable outcomes exist for cases of transplants performed inadvertently in patients with unrecognized pretransplant cryptococcosis and in those treated before transplantation [3].

However, a recent study by Singh *et al*. of cryptococcosis in patients with liver cirrhosis has provided us with new insights on this topic [9]. Of 112 patients with cryptococcosis and cirrhosis, 39 were deemed transplant candidates, and eight ultimately underwent transplantation. Two patients were still active on the list at the time of this review. In all, seven patients received liver transplants, and one received a double kidney-liver transplant. Of these eight patients, four had disseminated disease (including three with meningitis), two had pulmonary disease, and two had extra-pulmonary disease. All patients received tacrolimus-based immunosuppression, and three received induction therapy that included basiliximab in two liver transplant recipients and thymoglobulin in one liver-kidney transplant recipient. There was a survival benefit in favor of patients who were transplanted compared to patients who were not, with a 90-day mortality of 0% (0/8) *vs*. 61.3% (19/31), respectively. Overall, six of eight patients received antifungal therapy prior to transplantation, and all patients received prolonged suppressive therapy with fluconazole post-operatively. None of the transplanted patients had progression or recurrence of cryptococcosis [9].

Notably, two of these eight patients had active pre-transplant cryptococcal infection that was not recognized until the post-operative period. One of them had cryptococcal peritonitis, and the other had fungemia and meningitis. However, it was only in the post-operative course that pre-transplant cultures returned positive for *Cryptococcus*, and treatment was therefore not started until post-transplant day 1 and 2, respectively. Both patients received liver transplants, and neither of them received basiliximab induction. Both were at alive at 90 days with no relapse of cryptococcosis, however the patient with disseminated disease ultimately expired 249 days post-transplant for reasons unrelated to fungal infection [9].

Thus, cirrhotics with cryptococcosis may not be categorically excluded from transplantation. Rather, any stable cirrhotic with cryptococcosis may be cautiously transplanted, on a case-by-case basis [9].

## 3. Donor-Derived Cryptococcus

Cryptococcosis typically occurs late after transplantation, with a median time to onset of 16–21 months [15,16]. Primary infection is thought to be inhalational, and overt disease usually represents reactivation of latent infection in the setting of immunosuppression, similar to what is seen in other granulomatous diseases such as tuberculosis and histoplasmosis [5,17]. However, transmission of cryptococcosis via donor organs has been described and is a potentially significant complication after organ and tissue transplantation [15,18,19,20,21].

The most incontrovertible evidence of donor transmission of *Cryptococcus* to date has been in three recipients of cadaveric organ transplants from a donor with unrecognized cryptococcal meningoencephalitis [16]. The liver recipient developed disseminated cryptococcosis (involving the liver, spleen, and lungs) 14 days post-transplant. One kidney recipient developed disseminated cryptococcosis (fungemia and pneumonia) 16 days post-transplant, and the other kidney recipient developed cryptococcal meningitis 24 days post-transplant. Both kidney recipients recovered with antifungal therapy, but the liver recipient died of unrelated causes. It was later discovered that the organ donor had been on long-term corticosteroids for sarcoidosis; however, cryptococcosis was not suspected at the time of organ procurement. Autopsy done 30 days after her liver and kidneys were transplanted revealed *C. neoformans* meningoencephalitis. All recipient isolates of *C. neoformans* were identical on multilocus sequence typing [16].

Thus, early post-transplant cryptococcosis (<4 weeks after transplant) warrants consideration of donor transmission [15,16]. Cryptococcosis should also be suspected in potential donors who succumb to an undiagnosed neurologic illness, especially in the setting of immunosuppression. Furthermore, unusual sites of presentation, such as the transplanted organ as the sole site of involvement or isolation of this yeast from surgical sites should also raise suspicion of donor transmission [15]. Prompt notification of organ procurement agencies when donor-derived disease is suspected is crucial.

## 4. Immune Reconstitution Syndrome

The immune reconstitution syndrome (IRS) is an entity that has been observed across a wide range of immunosuppressed hosts [22]. It is believed to be due to a shift from an anti-inflammatory state toward a proinflammatory state as a result of reduction of immunosuppression and reversal of pathogen-induced immunosuppression upon the initiation of antifungal therapy [3]. *C. neoformans*-associated IRS typically presents as lymphadenitis, enhancing CNS lesions, increased intracranial pressure with aseptic meningitis, or skin or soft tissue lesions [22]. The rapid immune restoration that occurs in IRS can mimic worsening cryptococcal disease, with new pulmonary infiltrates, new or worsening CNS masses, new leptomeningeal enhancement, and new skin or osteoarticular lesions developing despite appropriate antifungal therapy [23]. However, while cryptococci may be visualized histologically, cultures are negative in IRS [22]. Further, data linking it to increased mortality has been conflicting [23,24]. The incidence of IRS among HIV-positive individuals with cryptococcal meningitis upon initiation of antiretroviral therapy (ART) ranges from 10%–42%, and an estimated 5%–14% of SOT recipients with cryptococcosis may develop IRS, typically between four and six weeks after initiation of antifungal therapy [4,23,24].

### 4.1. Immunology and Pathogenesis

**Figure 1:**
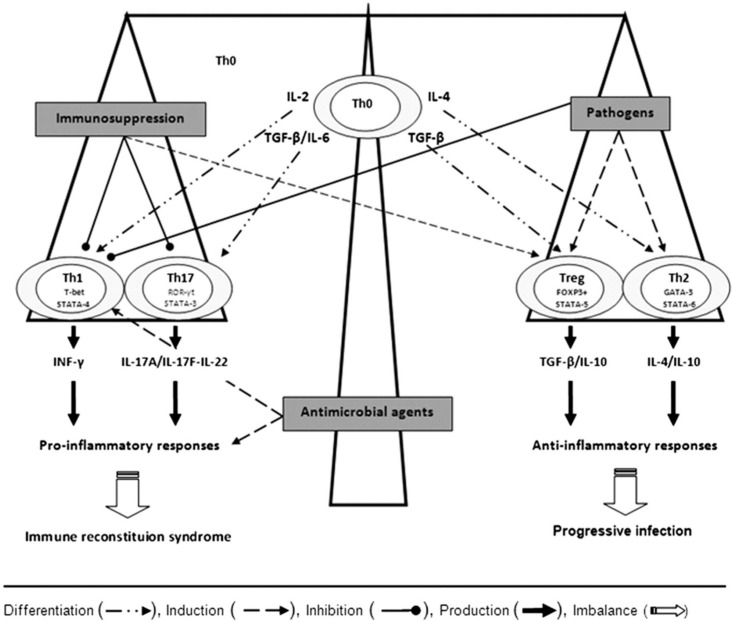
Depending on the cytokine milieu, the naïve or precursor T helper cells (Th0) differentiate into Th1, Th17, Treg, or Th2 cells via the expression of their specific transcription factors, T-bet/STAT-4, ROR-γt/STAT-3, FoxP3+/STAT-5, and GATA-3/STAT-6, respectively. Interferon IFN-γ and interleukin (IL)—17A/IL-17F/IL-22 by Th1 and Th17 cells, respectively, mediate inflammatory responses, whereas transforming growth factor (TGF)-β, IL-10, and IL-4 by T regulatory cells (Tregs) and Th2 cells lead to anti-inflammatory responses. The balance of these immune responses influences the development of immune reconstitution syndrome *versus* optimal pathogen eradication. Adapted from [26].

Antigen-presenting cells interact with naïve CD4+ T-helper (Th0) cells and cause them to differentiate into four functionally distinct subsets of cells based on the cytokine profile generated: Th1, Th2, Th17 (the effector cells) and T regulatory cells (Tregs) (Figure 1). Cytokines such as IL-12 and interferon (IFN)-γ stimulate the production of Th1 cells, which then release IL-2, tumor necrosis factor (TNF)-α, and IFN-γ. This results in potent pro-inflammatory responses, including the activation of macrophages that suppress intracellular infections [25]. Th2 cells do not generate inflammation, but instead promote the repair and recovery of tissues damaged by infection and activate cellular and antibody-mediated responses [25]. Th17 cells secrete proinflammatory cytokines (IL-17 and IL-6) that are responsible for the recruitment of neutrophils to infected tissue. Inappropriate production of IL-17 results in chronic inflammation and tissue destruction. Lastly, Tregs do not contribute to generation of the primary immune response to a pathogen, but help to restrain it once the pathogen poses no further threat [25]. The balance of these immune responses influences the development of IRS *versus* pathogen eradication [26].

A healthy immune response to cryptococcosis depends on the generation of proinflammatory Th1 and Th17 cells. In contrast, Th2 responses allow for proliferation of *Cryptococcus* within macrophages and thus facilitate dissemination [24]. In addition, the capsule of *C. neoformans* is immunomodulatory in its own right, preferentially stimulating a Th2 over Th1 response [8]. This state of pathogen-induced immunosuppression is potentially reversible upon initiation of antifungal therapy, resulting in a pronounced reversion from Th2 to Th1, with an exacerbation of inflammatory manifestations (Figure 1) [22,27]. Lastly, ineffective immune responses to *Cryptococcus* may occur in a subset of patients and may predispose to the development of IRS [24].

However, specific mechanistic studies for cryptococcal IRS have been performed almost exclusively in patients with HIV/AIDS and are lacking in SOT patients and other compromised hosts.

### 4.2. Cryptococcal IRS in HIV Infection

In a study of the pathogenesis of IRS, future cryptococcal-meningitis IRS was associated with increased pre-ART levels of Th2-mediated cytokines such as IL-4 and low levels of proinflammatory cytokines such as TNF-α, resulting in ineffective baseline immunity against *Cryptococcus* [24]. This is thought to result in impaired antigen recognition and clearance that may be conducive to IRS by allowing antigen persistence. Upon eventual immune restoration with ART, the excess uncleared cryptococcal antigen results in an intense cytokine storm and an exaggerated immune response (Table 1) [24]. Interestingly, CD4 count nadir, level of viremia, and rate of immune recovery after ART were not associated with IRS [24].

**Table 1:** Summary of paradoxical cryptococcal-IRS pathogenesis hypothesis in HIV. Adapted from [24].

Phase	Immunologic Activity	Evidence in CM-IRIS Patients
Before ART	Paucity of appropriate inflammation for cryptococcosis and/or	↓ TNF-α, G-CSF, GM-CSF, VEGF in serum ↓ IFN-γ, G-CSF, TNF-α, IL-6 in CSF
	Inappropriate (Th2) responses resulting in:	↑ IL-4 pre-ART
	Poor antigen clearance, pre-ART	Similar CSF CRAG at initial infection Higher CRAG pre-ART
After starting ART	Increasing proinflammatory signaling from APCs due to persisting antigen burden and failure to clear antigen	↑ IL6 from macrophages then downstream ↑ CRP production; ↑ IL-7 from APCs
	Secondary activation of coagulation cascade	↑ D-dimer
At IRIS	Cytokine storm of multiple immune pathways of innate and adaptive immune systems	Th1 ↑ INF-γ, VEGF; TH17 ↑ IL-17 Innate: ↑ IL-8, G-CSF, GM-CSF
	Activation of coagulation cascade	↑ D-dimer
	Neuronal cell activation and damage	↑ FGF-2

There is growing interest in the use of biomarkers to predict the occurrence of IRS. After initiation of ART, rising levels of C-reactive protein (CRP), D-dimer, and IL-6 were associated with a higher risk of developing IRS [24]. Further, persons who developed IRS after starting ART had a paucity of cerebrospinal fluid (CSF) inflammation at the time of initial diagnosis with cryptococcal meningitis compared with patients who did not develop IRS. The combination of initial CSF WBC count ≤25 cells/μL and CSF protein level ≤50 mg/dL was more highly associated with an increased risk of IRS [28]. Hypercalcemia has been reported in patients with IRS caused by cryptococcosis and may serve as a surrogate marker. Hypercalcemia is a recognized response to granulomatous disorders, with endogenous overproduction of 1,25-dihydroxyvitamin D by activated macrophages being the proposed mechanism [22]. However, whether any of these markers can be used to risk-stratify patients remains to be seen.

Lastly, the timing of ART during cryptococcal meningitis deserves special mention. While early initiation of ART in patients presenting with acute AIDS-related opportunistic infections results in better outcomes, conflicting data exists for cryptococcosis [2,29,30,31]. The recent Cryptococcal Optimal ART Timing (COAT) trial showed that deferring ART until five weeks after the start of amphotericin improved survival rates among patients with cryptococcal meningitis, as compared with initiating ART at one to two weeks [2]. Interestingly, earlier ART was most harmful in persons without CSF inflammation, and these patients developed higher CSF cellular infiltrate after ART was started. This suggests that the increased mortality from early ART was immunologically mediated [2,32]. In addition, there does not seem to be a benefit for earlier ART in treating tuberculous meningitis, implying that the timing of ART that provides the greatest advantage in patients with central nervous system (CNS) infections may differ from the timing in patients with non-CNS infections [2].

### 4.3. Cryptococcal IRS in Organ Transplant Recipients

The pathogenesis of cryptococcal IRS in SOT recipients is directly linked to their immunosuppression. Th1 and Th17 cells are the primary mediators of allograft rejection and are targets of immunosuppressive agents in transplant recipients, whereas Th2 cells and Tregs promote graft tolerance [26]. Calcineurin inhibitors preferentially suppress Th1 and Th17 and promote Th2 responses (Figure 1) [22,26]. Purine analogues, mTOR inhibitors, and corticosteroids also help foster an anti-inflammatory environment [22,26]. Thus, the cumulative effect of an immunosuppressive regimen in stable SOT patients reflects induction of tolerance by suppression of Th1 and Th17 cells and upregulation of Th2 cells, with or without Treg expansion [26].

Reduction or withdrawal of iatrogenic immunosuppression can therefore lead to a shift towards a proinflammatory phenotype by reversal of these responses [22]. In a study by Singh *et al*., only complete discontinuation of calcineurin inhibitors was independently associated with a 5-fold increased risk of IRS in SOT recipients with cryptococcosis [23]. In contrast, reduction in calcineurin inhibitor dose, discontinuation of prednisone, and discontinuation of azathioprine or mycophenolate mofetil were not associated with an increased risk of IRS [23].

Growing evidence suggests that immunodulatory characteristics of antifungal agents may also contribute to microbial pathogenesis. Although amphotericin B deoxycholate (AmBd) has exceptional anticryptococcal activity, it upregulates Th1 cells by Toll-like Receptor-2-mediated transcription of inflammatory cytokines [3,26]. It is plausible that, instead of attenuating, AmBd promotes the damage from excessive inflammation. Unlike AmBd, liposomal amphotericin B (L-AmB) either downregulates or has no effect on inflammatory cytokine gene expression. Thus, L-AmB may have a role in its anti-inflammatory properties [3,26]. Although intriguing, the clinical relevance of antifungal agent-associated immune modulation in the context of IRS remains to be fully defined.

### 4.4. Management of Cryptococcal IRS

Limited data are available that provide guidance on management of immunosuppression in SOT recipients with cryptococcosis. Withdrawal of immunosuppressive agents is intuitively logical but portends the risk of precipitating organ rejection and IRS. However, calcineurin inhibitors are not only protective against IRS, but they were also independently associated with a lower mortality in *C. neoformans* infection [23,33]. Furthermore, the literature suggests that calcineurin inhibitors have antifungal activity and offer synergistic interactions with antifungal agents [34]. Thus, the goal should be slow reduction but not abrupt cessation of calcineurin inhibitors, with consideration given to tapering corticosteroids first.

There is no proven treatment for established IRS. Minor manifestations such as lymphadenitis and skin lesions may resolve spontaneously, and modification of antifungal therapy is not warranted unless viable yeasts are isolated in culture [3]. Corticosteroids have been used with success in SOT recipients and may be considered for life-threatening manifestations or severe disease, particularly involving the CNS [3,4]. Because TNF-α plays a key role in the response against *Cryptococcus*, TNF-α inhibitors have been anecdotally used for IRS [3]. Infliximab was successfully used for IRS refractory to high-dose corticosteroids and cyclophosphamide in CNS tuberculosis [35]. A role for statins has been proposed since they promote Th2/Tregs, inhibit Th1, and block Th17 development, but this remains speculative [3].

## 5. Cryptococcal Antigen Screening

Because the incidence of cryptococcal disease in industrialized countries is relatively low, routine screening of asymptomatic HIV-infected individuals is not currently recommended by the 2009 United States Department of Health, nor is primary prophylaxis against *Cryptococcus* [36]. However, a different strategy may be required in resource-limited settings, where the incidence of asymptomatic cryptococcal antigenemia ranges from 3.8% to 21% [37]. Since a significant number of these patients initiate ART only after developing AIDS, they incur a risk of unmasking the subclinical cryptococcemia leading to a potentially catastrophic IRS event. Indeed, the unmasking of cryptococcal meningitis after initiating ART accounts for 30% of the cases of cryptococcal meningitis in Africa [37].

Thus, three strategies have been proposed to prevent cryptococcal-related mortality in patients with HIV in resource-poor settings: (1) initiation of ART prior to the development of AIDS, (2) primary prophylaxis with fluconazole in persons with AIDS, and (3) screening and treatment for occult cryptococcosis [37]. In a randomized controlled trial of HIV-positive Ugandan adults, primary prophylaxis with fluconazole 200 mg thrice weekly was safe and effective in preventing invasive cryptococcosis in patients with CD4+ counts ≤200 cells/μL and negative serum cryptococcal antigen [38]. Furthermore, screening for cryptococcal antigen in asymptomatic persons with a CD4+ cell count ≤100 cells/μL who were initiating ART, but were not receiving primary fluconazole prophylaxis, was cost-effective and prevented disease and death [37]. Subsequently, starting fluconazole in conjunction with ART upon detection of asymptomatic antigenemia prevented the development of cryptococcal meningitis [37].

In a cost-efficacy analysis from South Africa, cryptococcal antigen screening with targeted treatment proved practical and efficacious while still minimizing costs for patients with CD4+ counts ≤100 cells/μL. It was less expensive and more effective than both ART alone (with no screening or prophylaxis) and universal primary antifungal prophylaxis. When compared to cryptococcal antigen screening with subsequent lumbar puncture for those testing positive and treatment with either amphotericin B or fluconazole based on the presence or absence of CNS disease, a simple “screen and treat” strategy was only marginally less effective while still being considerably less costly [39]. Therefore, routine implementation of cryptococcal antigen screening in high-prevalence areas is rational.

While these interventions reduce *Cryptococcus*-related morbidity, there is conflicting data regarding their impact on survival [37,38,40]. In a Cochrane review, primary antifungal prophylaxis reduced the incidence of cryptococcosis but did not confer a clear mortality benefit [41]. However, the trials that were included were markedly heterogeneous. Similarly, a meta-analysis showed a reduction in *Cryptococcus*-specific mortality with primary prophylaxis but no impact on all-cause mortality [42].

Nonetheless, the above data are reflected in the World Health Organization (WHO) guidelines, which recommend screening for all ART-naïve patients with CD4 ≤100 cells/μL where the prevalence of cryptococcal antigenemia is >3%. This should be followed by preemptive antifungal therapy if the serum cryptococcal antigen is positive [43]. There is however no recommended antifungal prophylaxis against cryptococcosis in other hosts at risk for disease, including SOT recipients [4,44]. Additionally, the utility of routine screening of organ donors and recipients and assessment of cirrhotic patients for subclinical cryptococcal antigenemia before or during transplant candidacy is unknown.

## 6. *Cryptococcus gattii*

*C. neoformans* is distributed worldwide, and while *C. gattii* has long been an endemic pathogen in Australia, its epidemiology is changing. The spread of *C. gattii* into new geographic regions was heralded by an outbreak of infection on Vancouver Island in British Columbia, Canada, in 1999. It has subsequently become established in British Columbia and the Pacific Northwest of the United States, mainly in Washington and Oregon [45]. However, cases outside of areas of known *C. gattii* endemicity have been described worldwide [45,46]. There are four major molecular serotypes of *C. gattii*, designated VG1, VGII, VGIII, and VGIV, with different geographic distributions and degrees of virulence [45,46].

*C. gattii* has historically been known to cause disease in persons with apparently normal immune systems [45,46,47]. However, more recent data has led us to recognize that infection does occur in patients with overt immunocompromise [45,47]. Furthermore, subtle defects in phagocytic function may be found in patients with *C. gattii* infection who would otherwise be considered immunocompetent [45,47,48]. In a study of healthy individuals with CNS cryptococcosis, anti-granulocyte-macrophage colony-stimulating-factor (GM-CSF) autoantibodies were present in seven of nine patients with *C. gattii* infection but in none of those with *C. neoformans* infection [47]. These autoantibodies result in dysfunctional GM-CSF, leading to impairments in innate immunity, phagocytic activity, and Th1-cell responses [47]. Why this does not also predispose to *C. neoformans* is unknown.

*C. gattii* induces higher amounts of proinflammatory cytokines compared to *C. neoformans*, suggesting a more powerful immune response against this organism [49]. This may explain why pulmonary and cerebral cryptococcomas are much larger in *C. gattii* than *C. neoformans* and why devastating neurological sequelae are also more frequent in *C. gattii* infections [45,46]. IRS develops infrequently in *C. gattii*, but when it occurs in normal hosts, it is thought to be due to reversal of pathogen-induced immunosuppression via a shift from a *Cryptococcus*-induced Th2 response to a robust proinflammatory Th1 response with antifungal therapy [50].

Treatment of *C. gattii* infection is largely extrapolated from the experience with *C. neoformans* and from case series and expert opinion. The Infectious Diseases Society of America (IDSA) guidelines for cryptococcosis are the same regardless of the species, and while certain nuanced differences in management may exist, the general concepts and regimens remain similar [50,51]. Thus, routine identification of *Cryptococcus* to the species level is probably not necessary [49,52]. However, in contrast to *C. neoformans*, epidemiologic data suggests that some strains of *C. gattii* (particularly VGII) have relatively low susceptibilities to fluconazole, with sustained susceptibility to voriconazole and posaconazole [45]. This has led some to support the use of these extended-spectrum azoles during the continuation phase of treatment [46,49]. However, there are currently no Clinical and Laboratory Standards Institutes (CLSI) breakpoints for *C. gattii*, and further research into this is needed.

## 7. Conclusions

Since its discovery as an environmental yeast in 1894, *Cryptococcus* has emerged as a significant pathogen [5]. Its role in human disease is likely to increase as the immunocompromised population grows. Unlike several other mycoses, where available fungal markers are imperfect (e.g., *Aspergillus* galactomannan and β-d-glucan), the cryptococcal antigen assay is rapid, reliable, and highly accurate, with low rates of false positivity and false negativity [4]. Recent research has focused on nucleic-acid-based testing such as internal-transcribed spacer (ITS) sequencing for rapid diagnosis of *Cryptococcus*; their role in clinical practice however, remains to be determined [45]. In contrast to many filamentous moulds where drug treatments continue to garner controversy (e.g., the merit of dual antifungal therapy for invasive aspergillosis) or demonstrate suboptimal efficacy (e.g., *Scedosporidium*), treatment of *Cryptococcus* is largely standardized [53]. The advent of oral fluconazole in the 1990s with its excellent *in vitro* activity and low rates of resistance has been a major advance in the management of disease [51]. A challenging scenario however is patients with CNS disease who fail to eradicate the infection despite an appropriate course of induction therapy; their optimal management has not been fully defined [54]. Use of immunomodulatory therapies to augment a poor host response remains an area of ongoing interest.

Additionally, certain subgroups of patients continue to be at risk for poor outcomes, and their management remains suboptimal. Clinicians need to be aware of the propensity of cirrhotics to develop cryptococcosis and should consider early cryptococcal antigen testing in patients with ESLD and sepsis of undetermined etiology. Delays in administration of antifungal therapy in cirrhotics usually stemmed from a lack of awareness of their increased risk for cryptococcal infections and have contributed to mortality [10,14]. Whether asymptomatic cirrhotics will benefit from the “screen and treat” strategy that is currently recommended by the WHO for certain patients with AIDS is unknown, especially when considered from a pre-transplant perspective. Regardless, cryptococcal infection in a stable cirrhotic may not preclude transplantation.

Finally, there is growing recognition of IRS as a distinct entity in diverse hosts. Although ART has led to dramatic improvement in patients with AIDS, the precise timing of its initiation remains controversial given the risk of IRS. Based on emerging data however, deferring it until after the employment of antifungal therapy appears rational. Likewise, evidence-based data in organ transplant recipients has shown that continuation of calcineurin-inhibitor agents at any dose, as opposed to their discontinuation, is beneficial. Investigations for diagnostic markers to establish timely diagnosis of IRS and to differentiate it from disease progression remain an unmet need that warrants future assessment.

## References

[1] Kwon-Chung K.J., Fraser J.A., Doering T.L., Wang Z., Janbon G., Idnurm A., Bahn Y.S. (2014). *Cryptococcus neoformans* and *Cryptococcus gattii*, the etiologic agents of cryptococcosis. Cold Spring Harb. Perspect. Med..

[2] Boulware D.R., Meya D.B., Muzoora C., Rolfes M.A., Huppler Hullsiek K., Musubire A., Taseera K., Nabeta H.W., Schutz C., Williams D.A. (2014). Timing of antiretroviral therapy after diagnosis of cryptococcal meningitis. N. Engl. J. Med..

[3] Singh N. (2012). How I treat cryptococcosis in organ transplant recipients. Transplantation.

[4] Baddley J.W., Forrest G.N., The AST Infectious Diseases Community of Practice (2013). Cryptococcosis in solid organ transplantation. Am. J. Transplant..

[5] Perfect J.R., Mandell G.L., Bennett J.E., Dolin R. (2010). Cryptococcus neofomans. Mandell, Douglas, and Bennett’s Principles and Practice of Infectious Diseases.

[6] Pappas P.G., Perfect J.R., Cloud G.A., Larsen R.A., Pankey G.A., Lancaster D.J., Henderson H., Kauffman C.A., Haas D.W., Saccente M. (2001). Cryptococcosis in human immunodeficiency virus-negative patients in the era of effective azole therapy. Clin. Infect. Dis..

[7] Sun H.Y., Wagener M.M., Singh N. (2009). Cryptococcosis in solid-organ, hematopoietic stem cell, and tissue transplant recipients: Evidence-based evolving trends. Clin. Infect. Dis..

[8] Buchanan K.L., Murphy J.W. (1998). What makes cryptococcus neoformans a pathogen?. Emerg. Infect. Dis..

[9] Singh N., Sifri C.D., Silveira F.P., Miller R., Gregg K.S., Huprikar S., Lease E.D., Zimmer A., Dummer J.S., Spak C.W. (2015). Cryptococcosis in patients with cirrhosis of the liver and posttransplant outcomes. Transplantation.

[10] Jean S.S., Fang C.T., Shau W.Y., Chen Y.C., Chang S.C., Hsueh P.R., Hung C.C., Luh K.T. (2002). Cryptococcaemia: Clinical features and prognostic factors. QJM.

[11] Runyon B.A. (1988). Patients with deficient ascitic fluid opsonic activity are predisposed to spontaneous bacterial peritonitis. Hepatology.

[12] Such J., Guarner C., Enriquez J., Rodriguez J.L., Seres I., Vilardell F. (1988). Low C3 in cirrhotic ascites predisposes to spontaneous bacterial peritonitis. J. Hepatol..

[13] Casadevall A., Perfect J.R., Cassadevall A., Casadevall A., Perfect J.R. (1998). Physical defenses and nonspecific immunity. Cryptococcus Neoformans.

[14] Singh N., Husain S., De Vera M., Gayowaski T., Cacciarelli T.V. (2004). Cryptococcus neoformans infection in patients with cirrhosis, including liver transplant candidates. Medicine.

[15] Sun H.Y., Alexander B.D., Lortholary O., Dromer F., Forrest G.N., Lyon G.M., Somani J., Gupta K.L., del Busto R., Pruett T.L. (2010). Unrecognized pretransplant and donor-derived cryptococcal disease in organ transplant recipients. Clin. Infect. Dis..

[16] Baddley J.W., Schain D.C., Gupte A.A., Lodhi S.A., Kayler L.K., Frade J.P., Lockhart S.R., Chiller T., Bynon J.S., Bower W.A. (2011). Transmission of cryptococcus neoformans by organ transplantation. Clin. Infect. Dis..

[17] Husain S., Wagener M.M., Singh N. (2001). *Cryptococcus neoformans* infection in organ transplant recipients: Variables influencing clinical characteristics and outcome. Emerg. Infect. Dis..

[18] Beyt B.E., Waltman S.R. (1978). Cryptococcal endophthalmitis after corneal transplantation. N. Engl. J. Med..

[19] Kanj S.S., Welty-Wolf K., Madden J., Tapson V., Baz M.A., Davis D., Perfect J.R. (1996). Fungal infections in lung and heart-lung transplant recipients: Report of 9 cases and review of the literature. Medicine.

[20] De Castro L.E., Sarraf O.A., Lally J.M., Sandoval H.P., Solomon K.D., Vroman D.T. (2005). *Cryptococcus albidus* keratitis after corneal transplantation. Cornea.

[21] Ooi H.S., Chen B.T.M., Cheng H.L., Khoo O.T., Chan K.T. (1971). Survival of a patient transplanted with a kidney infected with *Cryptococcus neoformans*. Transplantation.

[22] Singh N., Perfect J.R. (2007). Immune reconstitution syndrome associated with opportunistic mycoses. Lancet Infect. Dis..

[23] Sun H.Y., Alexander B.D., Huprikar S., Forrest G.N., Bruno D., Lyon G.M., Wray D., Johnson L.B., Sifri C.D., Razonable R.R. (2015). Predictors of immune reconstitution syndrome in organ transplant recipients with cryptococcosis: Implications for the management of immunosuppression. Clin. Infect. Dis..

[24] Boulware D.R., Meya D.B., Bergemann T.L., Wiesner D.L., Rhein J., Musubire A., Lee S.J., Kambugu A., Janoff E.N., Bohjanen P.R. (2010). Clinical features and serum biomarkers in HIV immune reconstitution inflammatory syndrome after cryptococcal meningitis: A prospective cohort study. PLoS Med..

[25] Parham P. (2015). Chapter 8: T cell-mediated immunity. The Immune System.

[26] Sun H.Y., Singh N. (2011). Opportunistic infection-associated immune reconstitution syndrome in transplant recipients. Clin. Infect. Dis..

[27] Einsiedel L., Gordon D.L., Dyer J.R. (2004). Paradoxical inflammatory reaction during treatment of *Cryptococcus neoformans* var. *gattii* meningitis in an HIV seronegative woman. Clin. Infect. Dis..

[28] Boulware D.R., Bonham S.C., Meya D.B., Wiesner D.L., Park G.S., Kambugu A., Janoff E.N., Bohjanen P.R. (2010). Paucity of initial cerebrospinal fluid inflammation in cryptococcal meningitis is associated with subsequent immune reconstitution inflammatory syndrome. J. Infect. Dis..

[29] Zolopa A., Andersen J., Powderly W., Sanchez A., Sanne I., Suckow C., Hogg E., Komarow L. (2009). Early antiretroviral therapy reduces aids progression/death in individuals with acute opportunistic infections: A multicenter randomized strategy trial. PLoS ONE.

[30] Makadzange A.T., Ndhlovu C.E., Takarinda K., Reid M., Kurangwa M., Gona P., Hakim J.G. (2010). Early *versus* delayed initiation of antiretroviral therapy for concurrent HIV infection and cryptococcal meningitis in sub-saharan Africa. Clin. Infect. Dis..

[31] Bisson G.P., Molefi M., Bellamy S., Thakur R., Steenhoff A., Tamuhla N., Rantleru T., Tsimako I., Gluckman S., Ravimohan S. (2013). Early *versus* delayed antiretroviral therapy and cerebrospinal fluid fungal clearance in adults with HIV and cryptococcal meningitis. Clin. Infect. Dis..

[32] Scriven J.E., Rhein J., Hullsiek K.H., von Hohenberg M., Linder G., Rolfes M.A., Williams D.A., Taseera K., Meya D.B., Meintjes G. (2015). Early art after cryptococcal meningitis is associated with cerebrospinal fluid pleocytosis and macrophage activation in a multisite randomized trial. J. Infect. Dis..

[33] Singh N., Alexander B.D., Lortholary O., Dromer F., Gupta K.L., John G.T., del Busto R., Klintmalm G.B., Somani J., Lyon G.M. (2007). *Cryptococcus neoformans* in organ transplant recipients: Impact of calcineurin-inhibitor agents on mortality. J. Infect. Dis..

[34] Kontoyiannis D.P., Lewis R.E., Alexander B.D., Lortholary O., Dromer F., Gupta K.L., John G.T., Del Busto R., Klintmalm G.B., Somani J. (2008). Calcineurin inhibitor agents interact synergistically with antifungal agents *in vitro* against *Cryptococcus neoformans* isolates: Correlation with outcome in solid organ transplant recipients with cryptococcosis. Antimicrob. Agents Chemother..

[35] Arend S.M., Leyten E.M., Franken W.P., Huisman E.M., van Dissel J.T. (2007). A patient with de novo tuberculosis during anti-tumor necrosis factor-alpha therapy illustrating diagnostic pitfalls and paradoxical response to treatment. Clin. Infect. Dis..

[36] Kaplan J.E., Benson C., Holmes K.K., Brooks J.T., Pau A., Masur H., Centers for Disease Control and Prevention (CDC), National Institutes of Health, HIV Medicine Association of the Infectious Diseases Society of America (2009). Guidelines for prevention and treatment of opportunistic infections in HIV-infected adults and adolescents: Recommendations from CDC, the National Institutes of Health, and the HIV Medicine Association of the Infectious Diseases Society of America. MMWR Recomm. Rep..

[37] Meya D.B., Manabe Y.C., Castelnuovo B., Cook B.A., Elbireer A.M., Kambugu A., Kamya M.R., Bohjanen P.R., Boulware D.R. (2010). Cost-effectiveness of serum cryptococcal antigen screening to prevent deaths among HIV-infected persons with a CD4+ cell count ≤ 100 cells/microl who start HIV therapy in resource-limited settings. Clin. Infect. Dis..

[38] Parkes-Ratanshi R., Wakeham K., Levin J., Namusoke D., Whitworth J., Coutinho A., Mugisha N.K., Grosskurth H., Kamali A., Lalloo D.G. (2011). Primary prophylaxis of cryptococcal disease with fluconazole in HIV-positive ugandan adults: A double-blind, randomised, placebo-controlled trial. Lancet Infect. Dis..

[39] Jarvis J.N., Harrison T.S., Lawn S.D., Meintjes G., Wood R., Cleary S. (2013). Cost effectiveness of cryptococcal antigen screening as a strategy to prevent HIV-associated cryptococcal meningitis in South Africa. PLoS ONE.

[40] Butler E.K., Boulware D.R., Bohjanen P.R., Meya D.B. (2012). Long term 5-year survival of persons with cryptococcal meningitis or asymptomatic subclinical antigenemia in Uganda. PLoS ONE.

[41] Chang L.W., Phipps W.T., Kennedy G.E., Rutherford G.W. (2005). Antifungal interventions for the primary prevention of cryptococcal disease in adults with HIV. Cochrane Database Syst. Rev..

[42] Ssekitoleko R., Kamya M.R., Reingold A.L. (2013). Primary prophylaxis for cryptococcal meningitis and impact on mortality in HIV: A systematic review and meta-analysis. Future Virol..

[43] WHO (2011). Rapid advice: Diagnosis, Prevention and Management of Cryptococcal Disease in HIV-infected Adults, Adolescents and Children.

[44] Fischer S.A., Lu K., AST Infectious Diseases Community of Practice (2013). Screening of donor and recipient in solid organ transplantation. Am. J. Transplant..

[45] Chen S.C., Meyer W., Sorrell T.C. (2014). *Cryptococcus gattii* infections. Clin. Microbiol. Rev..

[46] Franco-Paredes C., Womack T., Bohlmeyer T., Sellers B., Hays A., Patel K., Lizarazo J., Lockhart S.R., Siddiqui W., Marr K.A. (2015). Management of *Cryptococcus gattii* meningoencephalitis. Lancet Infect. Dis..

[47] Saijo T., Chen J., Chen S.C., Rosen L.B., Yi J., Sorrell T.C., Bennett J.E., Holland S.M., Browne S.K., Kwon-Chung K.J. (2014). Anti-granulocyte-macrophage colony-stimulating factor autoantibodies are a risk factor for central nervous system infection by *Cryptococcus gattii* in otherwise immunocompetent patients. MBio.

[48] Rosen L.B., Freeman A.F., Yang L.M., Jutivorakool K., Olivier K.N., Angkasekwinai N., Suputtamongkol Y., Bennett J.E., Pyrgos V., Williamson P.R. (2013). Anti-GM-CSF autoantibodies in patients with cryptococcal meningitis. J. Immunol..

[49] Rolston K.V. (2013). Cryptococcosis due to *Cryptococcus gattii*. Clin. Infect. Dis..

[50] Chen S.C., Korman T.M., Slavin M.A., Marriott D., Byth K., Bak N., Currie B.J., Hajkowicz K., Heath C.H., Kidd S. (2013). Antifungal therapy and management of complications of cryptococcosis due to *Cryptococcus gattii*. Clin. Infect. Dis..

[51] Perfect J.R., Dismukes W.E., Dromer F., Goldman D.L., Graybill J.R., Hamill R.J., Harrison T.S., Larsen R.A., Lortholary O., Nguyen M.H. (2010). Clinical practice guidelines for the management of cryptococcal disease: 2010 update by the Infectious Diseases Society of America. Clin. Infect. Dis..

[52] Perfect J.R. (2012). The triple threat of cryptococcosis: It’s the body site, the strain, and/or the host. MBio.

[53] Martin-Pena A., Aguilar-Guisado M., Espigado I., Cisneros J.M. (2014). Antifungal combination therapy for invasive aspergillosis. Clin. Infect. Dis..

[54] Singh N., Lortholary O., Alexander B.D., Gupta K.L., John G.T., Pursell K.J., Munoz P., Klintmalm G.B., Stosor V., Del Busto R. (2005). Antifungal management practices and evolution of infection in organ transplant recipients with *C. neoformans* infection. Transplantation.

